# Differential Regulation of Matrix-Metalloproteinases and Their Tissue Inhibitors in Patients with Aneurysmal Subarachnoid Hemorrhage

**DOI:** 10.1371/journal.pone.0059952

**Published:** 2013-03-28

**Authors:** Marlene Fischer, Anelia Dietmann, Ronny Beer, Gregor Broessner, Raimund Helbok, Bettina Pfausler, Erich Schmutzhard, Peter Lackner

**Affiliations:** Department of Neurology, Innsbruck Medical University, Innsbruck, Austria; Charite Universitätsmedizin Berlin, Germany

## Abstract

**Background:**

Matrix metalloproteinases (MMPs) and their tissue inhibitors (TIMPs) are involved in vascular remodeling, (neuro)inflammation, blood-brain barrier breakdown and neuronal apoptosis. Proinflammatory mechanisms are suggested to play an important role during early brain injury and cerebral vasospasm after aneurysmal subarachnoid hemorrhage (SAH). This study aimed to analyze MMP-3, MMP-9, TIMP-1 and TIMP-3 in patients with SAH and their respective association with cerebral vasospasm (CVS).

**Methods:**

Blood samples were collected in 20 SAH patients on days 1 to 7, 9, 11, 13 and 15 and 20 healthy age and gender matched volunteers. Serum MMPs and TIMPs were analyzed using enzyme-linked immunosorbent assay. Doppler sonographic CVS was defined as a mean blood flow velocity above 120 cm/sec in the middle cerebral artery. When discharged from hospital and at 6 month follow-up neurological outcome was evaluated using the Glasgow Outcome Score and the modified Rankin Scale.

**Results:**

MMP-9 was higher in SAH patients compared to healthy controls (p<0.001). Patients with CVS (n = 11) had elevated MMP-9 serum levels compared to patients without CVS (n = 9, p<0.05). Higher MMP-9 was observed in the presence of cerebral ischemia associated with cerebral vasospasm (p<0.05). TIMP-1 was increased in patients with SAH on day 4 (p<0.05). There was an imbalance of the MMP-9/TIMP-1 ratio in favor of MMP-9 in SAH patients, in particular those with CVS (p<0.001). MMP-3 and TIMP-3 were significantly lower in SAH patients throughout day 4 and day 7, respectively (p<0.05). We did not find an association between MMP-, TIMP levels and neurological outcome after 6 months.

**Conclusions:**

MMP-3 and -9 are differentially regulated in SAH patients with both enzymes showing peak levels correlating with the development of CVS. The inhibitors TIMP-1 and -3 were low during the acute phase after SAH and increased later on which might suggest a preponderance of pro-inflammatory mechanisms.

## Introduction

Subarachnoid hemorrhage (SAH) accounts for 2–5% of all new strokes and is associated with high morbidity and mortality [1], [2]. Cerebral vasospasm (CVS), an important complication after aneurysmal SAH, may be associated with delayed cerebral ischemia contributing to poor functional outcome and death [3], [4], [5]. Recently, early brain injury during the first 72 hours after SAH, has been recognized as a crucial determinant of secondary brain damage [6], [7]. Moreover, it has been suggested that early brain injury contributes to the (later) development of cerebral vasospasm [6], [8], [9]. Matrix metalloproteinases-3 and-9 (MMP-3 and-9) are involved in remodeling of the extracellular matrix including degradation of the basal lamina and have been characterized as major players in (neuro)inflammation [10], [11]. Both, MMP-3 and MMP-9, contribute to vascular hyperpermeability and blood-brain barrier disruption [12], [13], [14]. Under inflammatory conditions increased release of MMP-9 from smooth muscle cells, infiltrating leukocytes and microglia contributes to endothelial and cellular damage and neuronal, glial and endothelial apoptosis [15], [16]. MMP-3 release is stimulated by the presence of proinflammatory cytokines including Tumor Necrosis Factor alpha and Interleukin-1β underlining its role in inflammation [17], [18]. In addition, MMP-3 has a crucial function in the regulation of neuronal apoptosis through acting on caspase-3 [19]. MMP activity is mainly controlled at the transcriptional level and modulated by their tissue inhibitors (TIMPs) [20]. Four members of the TIMP family have been described so far with varying affinity for single MMPs [20]. TIMP-1 is regarded as an inhibitor for both, MMP-3 and -9, playing an important role in inflammation [21], [22]. TIMP-3 has been recognized as a potent inhibitor of MMP-3 with mainly proapoptotic functions [23].

The aim of this study was to analyze the temporal profile of MMP-3, MMP-9, TIMP-1 and TIMP-3 serum levels in SAH patients and their association with cerebral vasospasm.

## Methods

### Ethics Statement

The study protocol was approved by the Ethics Committee at Innsbruck Medical University (Reference Number UN3021, 256/4.17).

### Study Population

Between November 2007 and January 2009 20 consecutive patients with aneurysmal SAH admitted to the neurocritical care unit of the Department of Neurology of Innsbruck Medical University were enrolled in this prospective pilot study. All patients were treated by endovascular coiling with electrolytically detachable platinum coils, six patients (30%) received additional vascular stents. Patients undergoing surgical clipping of aneurysms were not included due to potential effects of surgical trauma on MMP and TIMP serum levels. Inclusion criteria: SAH confirmed by cerebral computed tomography (CT), ruptured intracranial aneurysm demonstrated by digital substraction angiography (DSA) for which interventional coiling was possible, first signs and symptoms having occurred within 48 hours before screening, written informed consent before recruitment or at time of regaining consciousness and WFNS grades I-V. Exclusion criteria: intracerebral or intraventricular blood without aneurysmal bleeding source, moderate to severe vasospasm at screening angiography, known coagulopathies, treatment with thrombocyte aggregation inhibitors or vitamin-K antagonists and severe pre-existing concomitant diseases.

Twenty age and gender matched healthy volunteers were recruited from hospital workers and relatives of the study investigators (mean age: 52.2, range: 33–68). All data was analyzed on an intention-to-treat basis.

### Sample Collection and Measurement

Blood samples of SAH patients were prospectively collected daily for the first 7 days, then every other day until 15 days post SAH. The first sample was taken before DSA and coiling procedure. Single blood samples from 20 age and gender matched volunteer donors served as healthy controls. Blood was collected using Sarstedt Monovette serum tubes. After at least 30 minutes of clotting time serum was obtained by centrifugation at 1500 rcf for 15 min within two hours after blood collection and stored at -80°C until use. MMP-3, MMP-9, TIMP-1 and TIMP-3 were measured in serum samples using enzyme-linked immunosorbent assay (R&D Systems, Minneapolis, MN) according to the manufacturer’s instructions.

### Transcranial Doppler Sonography (TCD) and Patient Management

TCD was performed daily from day 1 to 7 and every other day thereafter. Recordings of the mean blood flow velocities (mBFV) were performed through the trans-temporal ultrasound window using a 2-MHz handheld transducer probe (Compumedics DWL Multidop X4, Melbourne, Australia) when pCO_2_ levels where within normal ranges. Doppler sonographic cerebral vasospasm (dCVS) was defined as mBFV of 120cm/s or more in the middle cerebral artery. DCI was defined as new infarct on CT scan that had not been detected on the admission or the immediate post-interventional scan, and that was classified as vasospasm related by the research team (cerebral ischemia attributable to vasospasm, CIV). Other potential causes of CT pathologies, (e.g. rebleeding, cerebral edema or ventriculitis) were excluded. CT scans were also performed at discharge and were assessed by an independent radiologist.

At the end of hospitalization and after 6 months outcome was evaluated by modified Rankin Scale (mRS) and the Glasgow Outcome Scale (GOS). Demographic, clinical and laboratory values were recorded prospectively throughout the study. Patients experiencing dCVS received hemodynamic augmentation involving a target central venous pressure of >8 mm Hg according to local protocols, which have been published previously [2]. Hypertension was induced using norepinephrine or phenylephrine infusion and fluid to maintain a mean arterial blood pressure of ≥100 mmHg. All patients received nimodipine either per os (target daily dose 300 mg) or intravenously (target daily dose 48 mg), unless hemodynamic instability occurred.

### Statistical Methods

MMP and TIMP levels were compared between the patient groups by Wilcoxon rank-sum test and corrected for multiple comparisons using the false discovery rate criterion (FDR) [24]. To test the association between cerebral vasospasm or cerebral ischemia and the levels of MMP-9, MMP-3, TIMP-1 and TIMP-3 corrected for important covariates (age, sex, white blood cell count (WBC), C-reactive protein (CRP) and body temperature), generalized estimating equations were calculated with day post SAH and presence of dCVS or cerebral ischemia as factors, respectively. To avoid co-linearity five different models were calculated, one for each of the respective covariates. Data are presented as mean ± SEM unless otherwise stated. Calculations were done using the PASW 18 (SPSS Inc., Chicago, IL, USA) and GraphPad Prism 5.00 software (GraphPad Prism Software Inc., San Diego, CA, USA).

## Results

### Patients’ Characteristics

Patients’ age ranged from 31 to 66 years (mean 52.2 years), 4 patients were male 16 female. One patient showed mild angiographic CVS during intervention on day one. Another ten patients developed Doppler sonographic CVS (dCVS) between day 2 and 13 (1 patient on day 2, 4 patients on day 4, 3 patients on day 6, 1 patient on day 11 and 1 patient on day 13). Seven patients developed cerebral ischemia attributable to vasospasm. Demographic, clinical and laboratory characteristics of all patients are listed in table 1 and were compared based on the presence of dCVS. Baseline characteristics were comparable between both groups.

**Table 1:** Baseline characteristics including demographic and laboratory data of the study population.

Parameter	dCVS absent	dCVS present	p-value
**number of patients**	9	11	
**age (mean, range)**	52.6 (31–66)	51.9 (39–64)	0.655*
**female gender, n (%)**	8 (88.9%)	8 (72.7%)	0.369†
**WFNS scale, n (%)**			0.119†
**I**	4 (44.4%)	3 (27.3%)	
**II**	0 (0%)	4 (36.4%)	
**III**	2 (22.2%)	0 (0%)	
**IV**	1 (11.1%)	3 (27.3%)	
**V**	2 (22.2%)	1 (9.1%)	
**modified Fisher scale, n (%)**			0.243†
**II**	2 (22.2%)	0 (0%)	
**III**	2 (22.2%)	4 (36.4%)	
**IV**	5 (55.6%)	7 (63.6%)	
**mRS on discharge**			0.545†
**0**	3 (33.3%)	4 (36.4%)	
**1**	3 (33.3%)	1 (9.1%)	
**2**	0 (0%)	1 (9.1%)	
**3**	1 (11.1%)	3 (27.3%)	
**4**	0 (0%)	1 (9.1%)	
**5**	1 (11.1%)	0 (0%)	
**6**	1 (11.1%)	1 (9.1%)	
**Length of stay in days, mean (range)**	20.3 (9–41)	25.5 (12–53)	0.394*
**Occlusive hydrocephalus requiring EVD**	3 (33.3%)	7 (63.6%)	0.178†
**Ventriculo-peritoneal shunt**	1 (11.1%)	1 (9.1%)	0.881†
**Intracerebral hemorrhage**	3 (33.3%)	3 (27.3%)	0.769†
**Cerebral edema**	5 (55.6%)	8 (72.7%)	0.423†

dCVS: Doppler sonographic cerebral vasospasm, WFNS: World federation of neurosurgical societies, mRS: modified Rankin scale, EVD: external ventricular drainage,

* Analysis of variance,

† chi-square test.

### Temporal Profile of MMP-3, MMP-9, TIMP-1 and TIMP-3 Serum Levels

**Figure 1:**
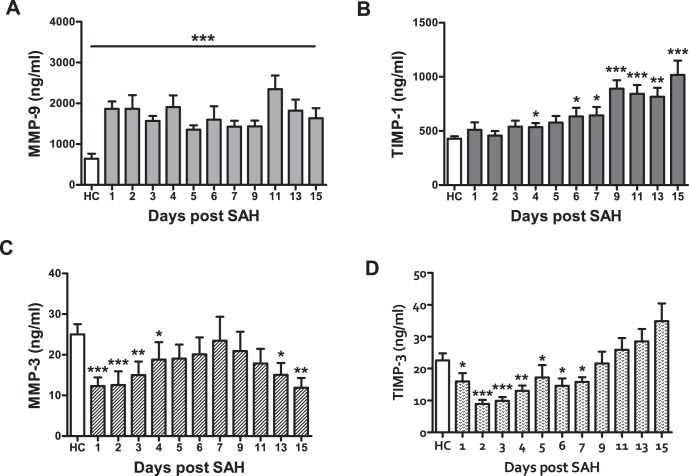
Matrix metalloproteinases (MMP) -3, -9, and their tissue inhibitors TIMP-1 and TIMP-3 in patients with subarachnoid hemorrhage (SAH). MMP-9 (A) is significantly elevated in SAH patients compared to healthy controls (HC). By contrast MMP-3 (C) is significantly lower during the early phase after SAH, but increases later on. Serum levels of both tissue inhibitors (B, D) show a delayed rise in SAH patients. * p<0.05, ** p<0.01, *** p<0.001.

Figure 1 shows the time course of MMP-3, MMP-9, TIMP-1 and TIMP-3 throughout day 15. Mean MMP-9 serum levels were significantly elevated in SAH patients compared with healthy controls (p<0.001). When comparing single days, MMP-9 was significantly higher in SAH patients compared with healthy subjects (p<0.001) on all study days.

TIMP-1 was significantly higher in SAH patients than in healthy subjects on days 4, 6, 7 (p<0.05) 9, 11, 13 and 15 (p<0.01). The MMP-9/TIMP-1 ratio was imbalanced in favor of MMP-9, which was mainly driven by the strong increase of MMP-9 in SAH patients.

MMP-3 was significantly lower in SAH patients on days 1, 2 (p<0.001), 3 (p<0.01), 4 (p<0.05) and days 13 (p<0.05) and 15 (p<0.01). Also TIMP-3 levels were decreased in SAH patients compared with healthy control subjects. There was a significant difference on days 1 (p<0.05), 2, 3 (p<0.001), 4 (p<0.01) and 5 to 7 (p<0.05). There was no statistically significant difference for days 9, 11, 13, and 15 (Figure 1D).

We did not find an association between MMP or TIMP levels and neurological outcome after 6 months as assessed by the Glasgow Outcome Score and the modified Rankin Scale.

### Doppler Sonographic Cerebral Vasospasm and Cerebral Ischemia

**Figure 2:**
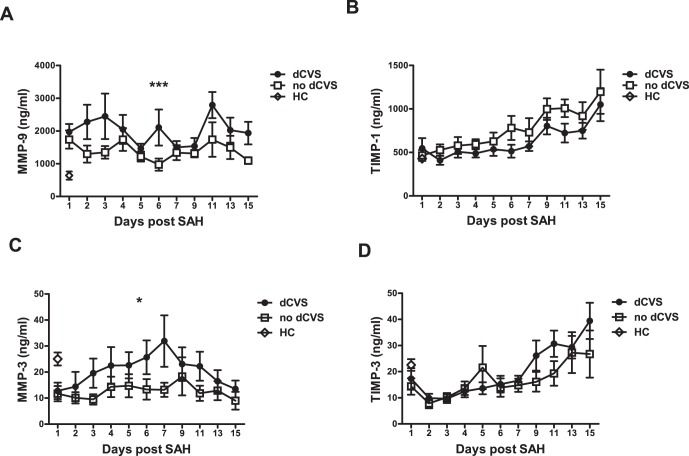
Matrix metalloproteinases (MMP) -3, -9, and their tissue inhibitors TIMP-1 and TIMP-3 in patients with Doppler sonographic cerebral vasospasm (dCVS) compared with patients without dCVS. MMP-9 (A) and MMP-3 (C) are significantly elevated in patients showing dCVS. * p<0.05, *** p<0.001.

To analyze the time course of MMP and TIMP levels and its association with the development of dCVS, multivariate generalized estimation equations were calculated with day post SAH and presence of dCVS as factors. This model showed a statistically significant effect of the interaction of both factors for MMP-9 (p<0.001) and MMP-3 (p<0.05) indicating different dynamics for MMP-9 and MMP-3 over time in patients with or without dCVS, respectively. In patients with cerebral vasospasm MMP-9 showed peaks during the early phase (day 1–3). In contrast, patients without dCVS lacked those MMP-9 elevations and revealed almost continuous MMP-9 levels. MMP-3, although decreased in SAH patients compared to healthy controls, was significantly higher in patients with dCVS peaking at day 7 slowly converging with the levels observed in patients without dCVS thereafter. MMP-3 remained continuously low in patients who did not develop dCVS throughout the first 14 days after bleeding. For TIMP-1 and TIMP-3 serum levels no significant association with dCVS was found. For detailed plotting see Figure 2.

In order to control the observed statistical association for important patient characteristics (age, sex) and variables associated with inflammation (WBC, CRP and body temperature) GEE models including these covariates were calculated. These models showed the same level of significance.

In line with the data for dCVS, a statistically significant effect of the interaction of factors cerebral ischemia and time for MMP-9 (p<0.05) was observed, which remained significant after inclusion of the above mentioned covariates. For MMP-3 and TIMP-3 a trend for the association with cerebral ischemia was observed (p = 0,06 and p = 0.054).

## Discussion

The main findings of this study are that: 1) MMP-9 and TIMP-1 serum concentrations are significantly higher in SAH patients compared to healthy controls, 2) serum levels of MMP-9 (in particular the MMP-9/TIMP-1 ratio) and MMP-3 are significantly elevated in patients with dCVS compared to patients without dCVS, 3) this divergence starts in the very early phase of disease showing strongly differing values already on the second day after bleeding, and 4) both, MMP-3 and TIMP-3 are significantly decreased during the first days after spontaneous SAH.

Numerous studies suggest a crucial role of MMP-9 in the pathophysiology of aneurysmal SAH [25], [26], [27], [28]. Downregulation of proinflammatory mediators - including MMP-9 - was found to reduce the cerebrovascular inflammatory response and late cerebral ischemia after experimental SAH [29]. MMP-9 is responsible for inactivation of plasma-type gelsolin, an anti-inflammatory mediator [30]. Decreased levels of plasma-type gelsolin have been found in SAH patients in combination with elevated cerebrospinal fluid levels of MMP-9 [25]. These findings suggest that one way of MMP-9 action in SAH is the inactivation of a sufficient anti-inflammatory response. Treatments aiming at the inhibition of neutrophil activity, including MMP-9 release, during the early phase after SAH have reduced microvascular injury and contributed to improved outcome after experimental SAH [31]. The pivotal role of MMP-9 during the acute phase after SAH is underlined by animal studies using the MMP-9 antagonist minocycline, which has been shown to improve outcome after SAH in rats [32], [33]. Changes of MMPs and TIMPs in our patients might be attributable to an increased pro-inflammatory state after acute SAH. Inflammation cascades consist of a variety of factors, MMPs and TIMPs mirroring only a minor aspect of these complex pathways. However, major parameters associated with inflammation including WBCs, CRP and body temperature, have been controlled for indicating a predominant role of MMP-9, -3, TIMP-1 and -3 in the pathophysiology after aneurysmal SAH.

Mean MMP-9 as wells as the MMP-9/TIMP-1 ratio were elevated in patients with dCVS in our study population. Data regarding the association of MMP-9 with the development of cerebral vasospasm are contradictory. Chou and colleagues did not find any association between MMP-9 levels and the occurrence of cerebral vasospasm [25]. They report an elevation of MMP-9 during the first days after SAH [25]. This is in contrast to our findings showing an increase of MMP-9 not only during the early phase after SAH, but also during later stages, when cerebral vasospasm has been found to be present. Interestingly, elevated levels of MMP-9 were not only associated with cerebral vasospasm in our study population but also with the presence of cerebral ischemia attributable to cerebral vasospasm. Yet, this association was not transferable to 6 month outcome. This might be a consequence of the limited number of patients with cerebral ischemia (n = 7) since an increase of MMP-9 has been described in numerous animal and clinical stroke studies underlining the importance of MMP-9 in cerebral ischemia [34], [35], [36], [37].

MMP-9 has a pivotal function as a cleavage molecule for a variety of proteins including the activation of Endothelin-1, which has been considered an important factor in the pathophysiology of cerebral vasospasm [38]. Interestingly, Endothelin-1 leads to increased production of MMP-3 in astrocytes [39]. In the present study patients with dCVS revealed significantly higher MMP-3 serum levels than patients without dCVS. Given an increased permeability of the blood-brain barrier in patients with SAH this elevation may be caused by astrocytic MMP-3 production in our study population correlating with the time at highest risk for CVS.

In contrast to the early up-regulation of MMP-9 in SAH patients compared to healthy controls, MMP-3 remained continuously lower. MMP-3 is known to activate MMP-9 [40]. Therefore, one possible explanation for the observed imbalance of MMP-3 and -9 might be a counterregulatory decrease of MMP-3 in response to overactivity of MMP-9. Similar dynamics have been described in patients suffering from ischemic stroke [41]. Notably, MMP-3 has been shown to be linked to coagulation [42]. Therefore, the sharp decrease of MMP-3 levels during the first days after SAH might be attributable to increased consumption of coagulatory factors, triggered by aneurysm rupture.

The expression of MMPs under physiologic conditions is mainly controlled at the transcriptional level [10]. A tight balance between (degrading) MMP activity and their endogenous tissue inhibitors is crucial [20]. TIMP-1 is highly inhibitory for MMP-9 [21]. We found a delayed increase of TIMP-1 in SAH patients starting on day 6 indicating an overshoot of MMP activity during the initial phase after SAH [43], [44]. This is the first study that investigates TIMP-1 serum concentrations in SAH patients so far. However, animal studies suggest an upregulation of TIMP-1 after experimental SAH [29], [45].

In line with the delayed increase of TIMP-1, TIMP-3 was significantly reduced in the acute phase after SAH. Since TIMP-3 has been shown to have anti-inflammatory effects [46] this down-regulation might corroborate the importance of pro-inflammatory mechanisms in the pathophysiology of SAH in general and for early brain injury in particular.

Our study, designed as a pilot study, only included a small number of patients, a potentially limiting factor. Importantly, the statistical models were corrected for important covariates and patients were well matched and showed a representative distribution of demographic and clinical characteristics. In addition the incidence of CVS, DCI and mortality was similar to previously local and international published data. However due to the low sample size our results will have to be verified in a larger patient sample.

To our knowledge this is the first longitudinal study focusing on MMPs and their respective tissue inhibitors in SAH. Early changes of MMPs and their tissue inhibitors were observed, they might play a role in the pathophysiology of acute SAH.
